# A Neuroendocrine–Immune Model of Hidradenitis Suppurativa: Mechanistic Insights into Pain, Pruritus, and Hormonal Triggers

**DOI:** 10.3390/jcm15103820

**Published:** 2026-05-15

**Authors:** Sophie M. Bilik, Rebecca E. Kaiser, Jacob Jalal Shawwa, Benjamin Fleischmann, Sierra Simecek, Irena Pastar, Rivka C. Stone

**Affiliations:** 1 Wound Healing and Regenerative Medicine Research Program, Dr. Phillip Frost Department of Dermatology and Cutaneous Surgery, University of Miami Miller School of Medicine, Miami, FL 33136, USA; rebeccakaiser@med.miami.edu (R.E.K.); jacobjshawwa@miami.edu (J.J.S.); srs416@med.miami.edu (S.S.); ipastar@med.miami.edu (I.P.); rivka.stone@med.miami.edu (R.C.S.); 2 Herbert Wertheim College of Medicine, Florida International University, Miami, FL 33199, USA; bmf146@miami.edu

**Keywords:** hidradenitis suppurativa, neuroendocrine, HPA axis, sex hormones, neuropeptides, chronic pain, pruritus, neuroimmune crosstalk, cortisol dysregulation, psychoneuroimmunology

## Abstract

**Background/Objectives:** Hidradenitis suppurativa (HS) is a chronic inflammatory skin disease traditionally viewed through a purely dermatologic lens. However, this perspective fails to explain stress-induced flares, menstrual cycle-linked exacerbations, and severe pain and itch disproportionate to visible cutaneous inflammation. This narrative review synthesizes evidence supporting a neuroendocrine–immune model of HS pathogenesis, with emphasis on mechanisms underlying pain and itch. **Methods:** A comprehensive literature search was conducted using PubMed, Scopus, and Web of Science databases (January 1990–September 2025) with terms including hidradenitis suppurativa, neuroendocrine mechanisms, HPA axis, sex hormones, neuropeptides, pain (including nociceptive and neuropathic pain, burning, and dysesthesia), and pruritus (itch). Eligible studies included peer-reviewed research examining hormonal, stress-related, or neuropeptide mechanisms in HS. Data were synthesized into thematic categories: endocrine influences, HPA axis function, neuropeptide signaling, immune crosstalk, and clinical implications. **Results:** Sex hormones promote follicular occlusion and modulate immune responses, explaining perimenstrual flares. Prolactin amplifies inflammation during stress through immune cell activation. Insulin resistance and adipokine imbalance create pro-inflammatory conditions. Chronic stress induces HPA axis dysfunction with cortisol resistance, exacerbating inflammation. Neuropeptides released from cutaneous nerves amplify immune activation and directly mediate pain and itch. These pathways establish self-perpetuating feedback loops wherein inflammation drives stress, and neuroendocrine dysfunction amplifies immune responses. **Conclusions:** HS represents a systemic disorder with a strong neuroendocrine–immune component, rather than a purely dermatologic condition. This framework supports multidisciplinary management integrating hormonal therapies, targeted immunomodulation, and stress reduction. Future research should characterize neuroendocrine biomarkers and test combination therapies targeting multiple system nodes.

## 1. Introduction

Hidradenitis suppurativa (HS) is a chronic, debilitating inflammatory skin disease affecting intertriginous areas [1]. However, the clinical burden of HS extends far beyond visible skin lesions. Pain is among the most debilitating of these symptoms, reported by patients as constant, severe, and often disproportionate to visible disease severity [2]. Patients experience severe, often intractable pain that persists even between flares and significantly impairs quality of life, with symptom exacerbations tied to hormonal fluctuations, particularly during psychological stress and menstrual cycles in women [3,4,5,6]. Although menstrual cycle-associated flares are specific to women of reproductive age, a population that accounts for the 3:1 female-to-male predominance observed in Western cohorts the neuroendocrine–immune mechanisms discussed in this review are broadly relevant to all patients with HS [7]. HS occurs in men, prepubertal children of both sexes, and postmenopausal women, indicating that sex hormones function as modulators rather than sole drivers of disease. Current models that view HS as purely a dermatologic disorder fail to account for the prominence of these symptoms, their fluctuation with patients’ stress and hormonal cycles, and their persistence despite control of visible inflammation [1,5,6,8,9]. These limitations necessitate a broader conceptual framework that integrates the neuroendocrine and immune systems.

The neuroendocrine–immune concept represents a paradigm shift in understanding HS pathogenesis. This framework recognizes that the nervous, endocrine, and immune systems do not function in isolation but rather engage in dynamic bidirectional communication that shapes disease expression and progression [6,10]. In HS, multiple lines of evidence suggest all three systems are intimately involved: menstrual cycle-linked flares point to hormonal influences, stress-induced exacerbations implicate neuroendocrine mechanisms, and persistent immune activation drives the characteristic inflammatory lesions [8,11,12,13]. The neuroendocrine–immune axis represents an underexplored but clinically relevant framework for understanding HS, offering new perspectives on disease mechanisms and potential therapeutic targets.

This review focuses specifically on pain, pruritus, and hormonal crosstalk in HS, as these symptoms represent the intersection of neuroendocrine and immune dysfunction. Pain and itch are not simply consequences of inflammation; they reflect active neuroimmune processes involving neuropeptide release, neurogenic inflammation, and peripheral sensitization [14]. Similarly, hormonal crosstalk represents a fundamental regulatory mechanism that modulates both immune responses and neural signaling. By examining how hormonal fluctuations, stress responses, and neuropeptide signaling converge to influence immune activation and sensory perception, we aim to provide a mechanistic understanding of symptom generation in HS and to identify novel opportunities for therapeutic intervention that address the full spectrum of symptoms that negatively impact patient quality of life.

## 2. Materials and Methods

### 2.1. Literature Search Strategy

This narrative review synthesizes the peer-reviewed literature on neuroendocrine–immune interactions in HS, with a focus on the roles of stress, hormones, and neuropeptides in pain and itch. A comprehensive search was performed using PubMed, Scopus, and Web of Science databases. The detailed search strategy, including Medical Subject Headings (MeSH) terms, keywords, inclusion/exclusion criteria, filters applied, and number of final studies included, are summarized in Table 1.

### 2.2. Eligibility and Selection Criteria

Included studies met the following criteria: (1) peer-reviewed primary research or reviews with a focus on neuroendocrine, hormonal, or neuropeptide mechanisms in HS or related chronic inflammatory conditions; (2) use of molecular, immunological, or clinical assays to investigate neuroendocrine–immune interactions; (3) studies involving human subjects or mammalian models relevant to HS pathophysiology. Exclusion criteria included studies with insufficient mechanistic detail, sole focus on surgical or antimicrobial management without neuroendocrine context, and isolated case reports unless mechanistically informative. Studies were not excluded based on participant sex. Where available, sex-specific findings were extracted; however, the review emphasizes shared neuroendocrine–immune mechanisms relevant to both men and women with HS, while distinguishing female-specific hormonal phenomena such as menstrual cycle-associated flares. A total of 73 studies were included in this review, as detailed in Table 1.

### 2.3. Data Extraction and Synthesis

Data extracted from eligible studies included hormonal measurements (sex hormones, prolactin, cortisol, metabolic hormones), neuropeptide expression, immune markers (cytokines, immune cell populations), pain and itch assessment tools, disease severity measures, study population characteristics (including sex where reported), and therapeutic interventions. Where available, specific assay methods (ELISA, immunohistochemistry, gene expression analysis) and clinical outcome measures were noted. Findings were synthesized into thematic categories including endocrine influences, HPA axis function, neuropeptide signaling, neuroimmune crosstalk, symptom mechanisms, biomarkers, and therapeutic interventions. Formal meta-analysis was not performed due to heterogeneity in study design, outcome measures, and reporting methods.

### 2.4. Ethics Statement

As this manuscript is a literature-based review and does not involve new research with human or animal subjects, ethical approval was not required.

### 2.5. Data and Material Availability

This review does not include original experimental data. All the supporting literature cited is publicly available through academic databases or open access sources. No proprietary datasets or materials were used. There are no restrictions on material or information availability associated with this publication. As this manuscript was designed as a narrative review rather than a systematic review, formal PRISMA-guided screening and risk-of-bias assessment were not performed. Nevertheless, predefined inclusion and exclusion criteria were used to improve transparency and thematic consistency of study selection.

## 3. Results

**Figure 1:**
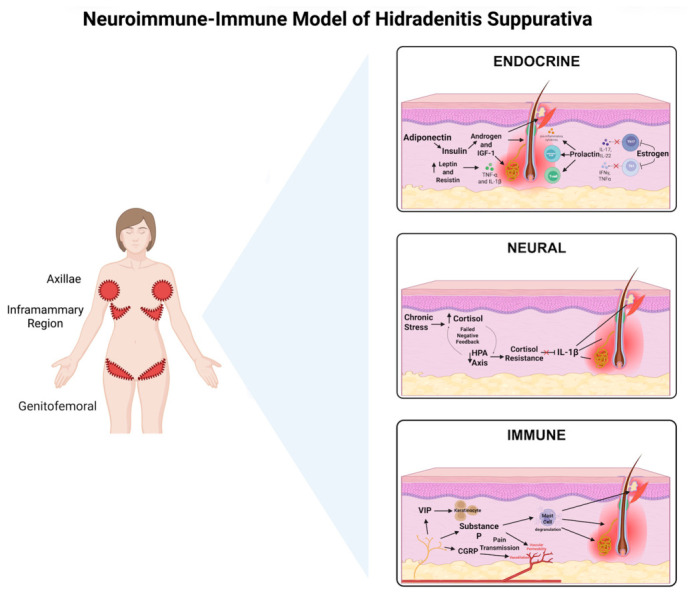
Neuroendocrine–immune model of hidradenitis suppurativa (HS). Schematic of the proposed neuroendocrine–immune framework underlying HS pathogenesis. The central diagram highlights common sites of disease involvement, including the axillae, inframammary, and genitofemoral regions. The right panels illustrate key interacting pathways. The endocrine panel shows hormonal and metabolic influences, including androgen signaling, insulin resistance, adipokine imbalance, and prolactin, contributing to follicular occlusion and inflammation. The neural panel depicts stress-induced HPA axis activation, impaired negative feedback, and cortisol resistance, leading to inadequate suppression of pro-inflammatory signaling (e.g., IL-1β). The immune panel highlights neuropeptide-mediated immune activation, including substance P, CGRP, and VIP, promoting mast cell activation, cytokine release, and nociceptor sensitization. HS; hidradenitis suppurativa; TNF-α; Tumor Necrosis Factor alpha; IL; Interleukin; CGRP; calcitonin gene-related peptide; SP; substance P; VIP; vasoactive intestinal peptide; Th; T helper; NK; Natural Killer; DC; Dendritic Cell; NLRP3; NOD-, LRR-, and Pyrin Domain-Containing Protein 3; NF-κB; Nuclear Factor kappa-light-chain-enhancer of activated B cells; AR; androgen receptor; IGF-1; insulin-like growth factor 1. HS; Hidradenitis suppurativa; TNF-α; Tumor Necrosis Factor alpha; IL; Interleukin; CGRP; calcitonin gene-related peptide; SP; substance P; VIP; Vasoactive IntestHidradenitis Suppurativa; TNF-α; Tumor Necrosis Factor alpha; IL; Interleukin; CGRP; calcitonin gene.

This review synthesizes evidence highlighting the complex interplay between neuroendocrine and immune systems in HS pathogenesis, with particular emphasis on pain and itch generation. Key dysregulated pathways include hormonal modulation of follicular occlusion and inflammation, HPA axis dysfunction with cortisol resistance, neuropeptide-mediated immune activation, and bidirectional neuroimmune feedback loops that perpetuate disease activity (Figure 1). The following subsections present findings from molecular, endocrinological, immunological, and clinical studies organized by mechanistic themes: endocrine influences, stress-axis dysfunction, neuropeptide signaling, immune crosstalk, and clinical implications for symptom management.

### 3.1. Endocrine Drivers of HS

Hormonal dysregulation plays a central role in HS pathogenesis by modulating follicular biology, immune activation, and inflammatory threshold. Androgens, prolactin, cortisol, and metabolic hormones influence not only lesion development but also the intensity of neuroimmune signaling that contributes to pain and pruritus [15,16,17,18]. Clinical observations of menstruation-related flares, pregnancy-associated changes, and metabolic comorbidities emphasize the importance of the endocrine system in HS. This section examines the mechanistic contributions of sex hormones to follicular dysfunction, the immune-modulatory effects of prolactin during stress, and the pro-inflammatory consequences of insulin resistance and adipokine imbalance.

#### 3.1.1. Sex Hormones and Reproductive Patterns

The predominance of HS in women of reproductive age, with a female-to-male ratio of approximately 3:1, further emphasize the role of sex hormones in disease pathogenesis [11]. In women with HS, flares often occur around menstruation, likely driven by cyclical hormonal shifts such as increased androgenic activity or end-organ hypersensitivity [11,18,19,20]. Perimenstrual worsening is reported by 43–77% of women, although a proportion experience no clear menstrual cycle-related symptom variation, suggesting heterogeneity in hormonal sensitivity rather than absence of hormonal involvement [11,19]. It is established that androgen receptor signaling promotes follicular hyperkeratosis and infundibular occlusion within the pilosebaceous-apocrine unit, a mechanism shared with acne vulgaris, lowering the threshold for follicular rupture and subsequent immune activation [5,8,19]. HS lesions show increased androgen receptor transcriptional activity and androgen-related genes are upregulated in HS patients [21]. Levels of sex hormone-binding globulin (SHBG) are reduced in obese patients, and this reduction can be seen in HS patients. Reduced SHBG levels increase circulating free androgens, which may amplify androgen-mediated follicular and immune effects [6]. Additionally, serum levels of pro-inflammatory adipokines, like omentin-1 and visfatin, are significantly elevated in HS patients compared to controls [5]. Anti-androgen therapies such as spironolactone and finasteride may benefit some women with mild-to-moderate disease [22]. Beyond androgen effects, estrogen can inhibit pro-inflammatory Th1 and Th17 cytokines, which may contribute to pregnancy-related improvements observed in some HS patients [11]. During pregnancy, responses are highly variable: approximately one-third of patients improve, one-third worsen, and one-third remain stable, with over half reporting symptoms resolving postpartum [11,19]. Women experiencing perimenstrual flares are more likely to improve during pregnancy; disease activity in these patients is sensitive to fluctuations in sex hormone balance and particularly shifts toward estrogen-dominant states [11]. After menopause, symptoms follow a variable course: most women report either worsening (39.5%) or no change (44.2%), while a minority experience improvement, indicating that menopause does not reliably induce remission [19]. This persistence after menopause, combined with HS occurrence in men and prepubertal children, indicates that sex hormones function as modulators of inflammatory intensity rather than sole drivers of disease, interacting with innate immune dysregulation and genetic susceptibility to shape overall disease phenotype.

#### 3.1.2. Prolactin and Stress-Hormone Interactions

Prolactin is a stress-responsive pituitary hormone with immunomodulatory properties that may contribute to HS disease activity by promoting immune activation and inflammation, particularly during stress and hormonal dysregulation [23]. Evidence directly assessing prolactin in HS patients is limited and heterogeneous. In women with premenstrual exacerbation of HS, endocrine stimulation testing demonstrated a functional hypothalamic–pituitary axis abnormality, with significantly exaggerated prolactin and thyroid-stimulating hormone (TSH) responses to combined thyrotropin-releasing hormone and gonadotropin-releasing hormone administration compared with controls [24]. These findings suggest altered hypothalamic–pituitary responsiveness in a subset of hormonally sensitive patients. In contrast, basal prolactin levels were not consistently elevated in general HS populations, suggesting that abnormalities may be stimulus-dependent rather than reflected at rest. During stress, reduced hypothalamic dopamine, normally an inhibitor of prolactin, leads to elevated prolactin levels [25]. Prolactin activates autoreactive B cells, modulates T cell responses, and amplifies pro-inflammatory cytokine production, thereby potentially intensifying inflammatory signaling in susceptible individuals [23,26]. In total, 672 HS cases across eight studies reported clinical improvement during pregnancy in 24% of patients, disease worsening in 20%, and postpartum flares in 60% of patients [27]. Notably, while prolactin levels are elevated in both pregnancy and the postpartum period, HS activity appears more closely associated with the postpartum hormonal milieu, characterized by persistently elevated prolactin in the setting of declining estrogen levels, rather than prolactin elevation alone [27]. Given that the postpartum period is characterized by persistently elevated prolactin levels with a rapid decline in estrogen, these findings suggest an association between prolactin-dominant hormonal states and HS disease activity, although causality remains unproven (28128074). Prolactin also alters disease expression through sebaceous and apocrine gland function by increasing sebum production, influencing keratinocyte biology, and disrupting glandular secretions, which can contribute to follicular blockage [23,28].

#### 3.1.3. Metabolic Hormones and Inflammation

Insulin resistance is two to three times more prevalent in HS patients than in matched controls and is independent of BMI, age, and sex [5,29,30]. In one cohort, HS patients demonstrated significantly elevated median Homeostatic Model Assessment of Insulin Resistance (HOMA-IR) values (2.0 vs. 1.5 in controls, *p* = 0.01) and a nearly threefold higher prevalence of insulin resistance (43.4% vs. 16.4%, *p* = 0.001) [29]. Although not directly associated with disease severity, it is a common comorbidity that may contribute to the chronic nature of HS [29,30]. By promoting a pro-inflammatory environment and disrupting adipokine balance, insulin resistance triggers immune activation and compromises epidermal barrier function [29]. Mechanistically, hyperinsulinemia can increase androgen production and enhance insulin-like growth factor-1 (IGF-1) signaling, both of which promote follicular hyperkeratosis and sebaceous gland hyperactivity, key early events in HS pathogenesis [11]. Obesity, highly prevalent in HS, represents a critical endocrine amplifier of this process. Excess adipose tissue functions as an active immune–endocrine organ, secreting adipokines that link metabolic dysfunction to sustained inflammatory signaling [5,31,32]. HS patients exhibit a characteristic adipokine profile with elevated pro-inflammatory mediators such as leptin and resistin, alongside reduced anti-inflammatory adiponectin [31,33,34]. These adipokines modulate both innate and adaptive immune responses, reinforcing Th1/Th17 polarization and cytokine production. Low adiponectin further worsens insulin resistance and systemic inflammation [32]. Elevated leptin and resistin promote innate and adaptive immune activation, leading to increased production of cytokines such as TNF-α and IL-1β, which are key drivers of HS inflammation [3,9,31,32] (Figure 1). Smoking, another major modifiable risk factor for HS, has been associated with systemic inflammation and insulin resistance in both general and HS-specific populations [35]. While direct mechanistic studies linking smoking-induced insulin resistance to HS pathogenesis remain limited, tobacco exposure may further exacerbate metabolic and inflammatory dysregulation in susceptible individuals. Chronic inflammation, in turn, further impairs insulin signaling, which may explain why metabolic interventions such as Glucagon-Like Peptide-1 (GLP-1) receptor agonists [36,37] benefit some HS patients beyond simple weight reduction.

### 3.2. The Hypothalamic–Pituitary–Adrenal (HPA) Axis

The HPA axis represents a critical link between psychological stress and physiological inflammation in HS. Chronic activation of this neuroendocrine pathway leads to paradoxical cortisol dysregulation, where persistently elevated cortisol levels fail to exert anti-inflammatory effects due to receptor resistance and impaired negative feedback [3,13,38,39,40]. This section examines the normal stress response physiology, the consequences of chronic HPA axis activation, evidence for cortisol resistance in chronic inflammatory diseases, and the specific hypothesis that HS-driven inflammation perpetuates maladaptive HPA axis feedback loops.

#### 3.2.1. Chronic Stress, HPA Axis Dysregulation, and Inflammation

The perception of stress triggers a set of physiological responses aimed at survival, prominently featuring sympathetic activation and the HPA axis [41]. Corticotropin-releasing hormone (CRH) released from paraventricular hypothalamic neurons stimulates pituitary adrenocorticotropic hormone (ACTH) secretion, which binds melanocortin 2 receptors (MC2R) in the adrenal zona fasciculata to drive cortisol synthesis and systemic release [42]. While acute stress responses are adaptive, chronic stress fundamentally alters this system through increased androgen sensitivity to ACTH, elevated baseline cortisol loss due to loss of negative feedback control, and sustained sympathetic and HPA axis activation [42,43]. This prolonged cortisol exposure leads to HPA axis desensitization and the development of cortisol resistance [28,29,34]. The resulting dysregulation disrupts the normal counterbalancing of pro-inflammatory mediators like IL-1β by melatonin and cortisol, creating a pathological imbalance between endocrine, nervous, and immune systems [44]. Similar HPA axis dysfunction is observed in other chronic inflammatory conditions including Alzheimer’s disease, Parkinson’s disease, depression, rheumatoid arthritis, and Crohn’s disease [44,45,46]. Consequently, chronic stress creates a paradoxical state where cortisol, normally anti-inflammatory, fails to suppress inflammation [47].

#### 3.2.2. HPA Axis Dysfunction in HS

Chronic stress in HS patients leads to increased cortisol production, which exacerbates skin inflammation [6,9,48]. Persistent inflammatory signaling and psychosocial stress promote sustained activation of CRH and ACTH, resulting in chronically elevated cortisol levels. As a result of chronic inflammation, the HPA axis fails to respond properly to cortisol resulting in persistently elevated cortisol levels due to failed negative feedback [9,48,49]. This maladaptive state resembles glucocorticoid resistance described in other chronic inflammatory diseases, in which prolonged cortisol exposure downregulates glucocorticoid receptor responsiveness and uncouples cortisol signaling from anti-inflammatory control [50]. Despite the elevated cortisol levels, cortisol fails to perform its anti-inflammatory function ultimately perpetuating the symptoms of HS [8]. These studies are further complicated by the circadian rhythm of cortisol levels. Although direct longitudinal studies examining systemic cortisol rhythms in HS remain limited, HS is consistently associated with high psychosocial stress burden and inflammatory cytokine activity, both of which can impair glucocorticoid receptor signaling. Pro-inflammatory mediators such as IL-1β and TNF-α have been shown to interfere with glucocorticoid receptor sensitivity, potentially contributing to functional cortisol resistance within lesional tissue [38,49,51]. This impaired glucocorticoid signaling may reduce the skin’s ability to terminate inflammatory cascades, thereby sustaining lesion activity and amplifying neurogenic inflammation. Psychosocial stress activates the systemic HPA axis, triggering hypothalamic CRH release, pituitary ACTH secretion, and adrenal cortisol production, which exerts both immunosuppressive and pro-inflammatory effects depending on magnitude and chronicity [52]. Importantly, human skin expresses a functional cutaneous HPA axis, including CRH, proopiomelanocortin (POMC), ACTH, and local cortisol synthesis, allowing cutaneous stress and inflammation to directly amplify immune activation [53,54]. In HS, where chronic barrier disruption and persistent cytokine signaling are present, local cutaneous HPA activation may reinforce rather than suppress inflammatory pathways. Psychosocial stress activates the systemic HPA axis, triggering hypothalamic CRH release, pituitary ACTH secretion, and adrenal cortisol production, which exerts both immunosuppressive and pro-inflammatory effects depending on magnitude and chronicity. Experimental models further demonstrate bidirectional skin–brain communication, whereby activation of the cutaneous HPA axis can signal centrally to reinforce systemic HPA activation [55]. In this context, HS may represent a reciprocal stress–inflammation loop in which immune activation drives stress signaling, stress enhances neuroendocrine dysregulation, and impaired cortisol responsiveness perpetuates inflammation. This cycle provides a mechanistic explanation for stress-induced flares and may contribute to the disproportionate pain and pruritus experienced by patients despite apparent systemic cortisol elevation.

### 3.3. Neuropeptides and Neurogenic Inflammation

In HS, neuropeptides represent a plausible mechanistic link between neurogenic inflammation and multiple disease domains [56]. Within the neuroendocrine–immune framework proposed in this review, neuropeptides are predicted to impact three primary domains: (1) pain generation, (2) pruritus (itch) signaling, and (3) immune amplification. Through stress-responsive release from cutaneous nerve fibers, neuropeptides may also indirectly influence endocrine signaling by reinforcing HPA axis activation. Given that HS patients experience severe pain with neuropathic characteristics and pruritus [57], neuropeptides may directly amplify inflammation and immune activation, contribute to pain and mediate pruritus. Substance P, calcitonin gene-related peptide (CGRP), and vasoactive intestinal peptide (VIP) are released from cutaneous nerve endings in response to stress and tissue damage, where they activate immune cells, promote vascular changes, and amplify inflammation [56]. The presence of neuropathic pain characteristics in HS suggests that neuropeptide-mediated neurogenic inflammation may contribute to the complex pain phenotype observed in these patients [57]. While the brain–skin axis provides a mechanistic framework for stress-induced HS flares, direct investigation of neuropeptide expression in HS lesions remains limited [10]. This section examines the known mechanisms of neuropeptide-mediated immune activation, the neural pathways linking psychological stress to cutaneous inflammation, and the significant research gaps that must be addressed to fully understand neurogenic contributions to HS pathogenesis.

#### 3.3.1. Neuropeptide-Mediated Immune Activation

Neuropeptides represent critical mediators in the neuroimmune interface, with substance P, calcitonin gene-related peptide (CGRP), and vasoactive intestinal peptide (VIP) playing particularly important roles in the generation of pain and pruritus in HS. Within the framework of this review, neuropeptides are positioned primarily as drivers of symptom expression, while simultaneously amplifying local immune activation [56]. These neuropeptides are released from peripheral nerve endings in response to stress and local inflammatory signals, exerting diverse effects on mast cells, vasculature, and keratinocytes [56]. Substance P promotes mast cell degranulation and enhances vascular permeability; CGRP modulates vasodilation and pain transmission; VIP influences immune cell function and keratinocyte proliferation [56]. Through direct sensitization of nociceptors and pruriceptors, substance P and CGRP can intensify pain signaling, while mast cell activation and neurogenic inflammation contribute to itch generation. The presence of neuropathic pain characteristics and pruritus in HS patients suggests these neuropeptides pathways may be active contributors to disease symptomatology [57]. Psychological stress activates both central and peripheral neural pathways that directly influence cutaneous immunity [58,59,60,61]. The brain–skin axis involves descending neural signals that trigger neuropeptide release from cutaneous nerve endings, creating a direct link between emotional states and local inflammatory responses. This stress-responsive neuropeptide release provides a plausible mechanistic basis for stress-induced HS flares and for the amplification of pain and pruritus during periods of heightened psychological distress [2,62].

#### 3.3.2. Cytokine and Immune-Mediated Mechanisms of Pain and Pruritus

In beginning stages of lesion formation, occluded hair follicles release damage-associated molecular patterns (DAMPs) and pathogen-associated molecular patterns (PAMPs). Nearby resident immune cells with receptors for DAMPs and PAMPs are activated to release pro-inflammatory cytokines like TNF and IL-1β [7,63,64]. As a result, local keratinocytes, endothelial cells, and fibroblasts are triggered to release chemokines that allow for the extravasation of neutrophils and other immune cells from the blood into skin [7,63,64]. IL-17 is also important for the entry of neutrophils into the skin [65]. IL-1β induces the production of metalloproteinases which allow for tunnel formation [66]. Additionally, IL-1β activates cytokines such as IL-6, IL-36 and G-CSF. IL-1β, IL-6, and TNF-α have all been implicated in the cause of pathological pain [67]. IL-1β has also been shown to increase levels of substance P and PGE2 also contributing to pain [67]. Mast cells have been implicated as the cause of itch in HS. Lesional skin contains significantly more mast cells than control skin [3]. Furthermore, it has been found that increased mast cells correlate with increased HS activity. Vossen et al. found evidence of perineural infiltrates composed of neutrophils and lymphocytes in lesional and perilesional skin [68]. Furthermore, they found evidence of neurogenic scar formation which they cited as a contributor to the itch experienced in HS patients [68]. Seventy-five percent of patients with HS experience both pain and itch, and since the C-fibers carry both the information for itch and pain via the lateral spinothalamic tract, patients may not be able to distinguish between the sensations.

#### 3.3.3. Research Gaps in Neuropeptide Expression in HS

While direct data on neuropeptide expression specifically in HS lesions remains limited, insights from other chronic inflammatory skin conditions, such as psoriasis and atopic dermatitis, suggest that neuropeptides play a significant role in disease activity [10,63,69]. In these conditions, neuropeptide concentrations within cutaneous nerve fibers and affected tissue are associated with clinical severity including pain and pruritus intensity and can be modulated by psychological stress, contributing to neurogenic inflammation and symptom exacerbation [63,64,65]. The paucity of neuropeptide data in HS represents a significant research gap and demonstrates the need for targeted investigations into neurogenic inflammation in this disease. Such studies could reveal novel therapeutic targets and biomarkers for disease monitoring. Future research should employ immunohistochemical analysis of substance P, CGRP, and VIP in lesional versus perilesional and non-lesional HS skin. Prior work in psoriasis has demonstrated the feasibility and relevance of this approach, with altered cutaneous neuropeptide expression identified through immunohistochemical staining [66]. Building on these findings, future studies in HS should aim to correlate neuropeptide expression with clinical measures of pain and pruritus, and to evaluate whether stress-reduction interventions can modulate cutaneous neuroimmune signaling in affected patients.

### 3.4. Neuroendocrine-Immune Crosstalk

While the preceding sections have examined sex hormones, metabolic hormones, stress responses, and neuropeptides as individual pathways, the pathogenesis of HS cannot be fully understood by examining hormonal, neural, or immune mechanisms in isolation. Rather, bidirectional communication between these systems creates integrated networks that shape disease presentation and progression. Hormones modulate immune cell function and trafficking, while inflammatory cytokines influence endocrine signaling and neuropeptide release [5,8,12]. Stress-induced neuropeptides activate immune cells, which in turn release mediators that sensitize sensory neurons and perpetuate pain and itch [10,67,68]. This section examines how hormonal and neuropeptide signals regulate both innate and adaptive immunity in HS, and how the resulting inflammatory milieu establishes self-perpetuating feedback loops that maintain chronic disease activity and resist therapeutic intervention.

A critical aspect of the neuroendocrine–immune interaction in HS is the establishment of self-perpetuating feedback loops. HS lesions generate pain and psychosocial stress, which activate the HPA axis and trigger neuropeptide release from cutaneous nerve endings [9,70]. As previously discussed, these neuropeptides and stress hormones then amplify immune activation, promoting further lesion formation and symptom generation. This vicious cycle explains the progressive nature of HS and the difficulty many patients experience in achieving disease control. Breaking this cycle requires interventions that target multiple nodes within the neuroendocrine–immune network. The chronicity of inflammation further drives HPA axis dysregulation and cortisol resistance, creating a state where the body’s natural anti-inflammatory mechanisms become ineffective [9,13].

Understanding these interconnected pathways is essential for developing rational combination therapies that address the systemic nature of HS rather than targeting isolated inflammatory mediators.

### 3.5. Clinical Implications of the Neuroendocrine–Immune Model in HS

The neuroendocrine–immune framework for HS pathogenesis has direct translational relevance for clinical practice, offering new approaches to diagnosis, risk stratification, and therapeutic intervention. Recognition that HS involves systemic hormonal dysregulation, stress-axis dysfunction, and neurogenic inflammation expands the therapeutic landscape beyond traditional immunosuppression and surgical management. This framework is particularly relevant for addressing the cardinal symptoms of pain and pruritus, which reflect integrated neuroimmune signaling rather than inflammation alone. Pain is among the most debilitating symptoms of HS, reported by patients as constant, severe, and often disproportionate to visible disease severity [2]. HS-associated pain demonstrates characteristics of both nociceptive pain (driven by tissue inflammation and damage) and neuropathic pain (resulting from nerve sensitization and dysfunction), with approximately 30% of patients exhibiting a high probability of neuropathic pain features such as burning, shooting, or electric shock-like sensations [57]. The intensity of pain in HS significantly impacts quality of life, work productivity, and psychological well-being [2]. Pruritus is a frequent but underreported symptom in HS patients, with moderate to severe intensity that significantly impacts daily activities and impairs quality of life [71]. Pruritus intensity correlates with disease activity and pain severity [57]. The mechanisms underlying both pain and pruritus in HS likely involve neuropeptide-mediated neurogenic inflammation, immune cell-derived mediators that sensitize sensory neurons, and potentially structural nerve damage from chronic inflammation and tissue destruction [72]. This section examines potential biomarkers of neuroendocrine dysfunction that may predict flares or treatment response. It also reviews current and emerging therapies targeting hormonal and stress pathways, while elucidating the mechanistic links between neuroendocrine dysregulation and the cardinal symptoms of pain and itch that define the HS patient experience. These therapeutic strategies, mapped to their respective neuroendocrine, immune, and neural targets, are summarized in Table 2.

#### 3.5.1. Biomarkers of Pain in HS Neuroendocrine Dysfunction

The circadian rhythm influences pain perception, with pain sensitivity exhibiting diurnal variation across the day. Twenty-two genes associated with the circadian rhythm linked to pain regulation were dysregulated in patients with HS [62]. These findings suggest that central neuroendocrine regulation of pain processing may be altered in HS, consistent with the stress axis and hormonal mechanisms discussed above. Additionally, HS patients exhibit dysregulated POMC gene, which is responsible for the production of beta-endorphins and ACTH [62]. This dysregulated POMC gene can result in abnormal beta-endorphin levels, which can greatly affect pain sensitivity and inflammation in patients with HS Additionally, dysregulation of CYP19A1 (aromatase) leads to altered estrogen balance, which can influence pain perception [62]. Further supporting the hormonal modulation of pain in HS, lesional tissue demonstrates increased androgen receptor transcriptional activity and reduced SHBG levels, which may further modulate inflammatory intensity and symptom expression, although their direct correlation with pain severity requires further investigation [18]. Taken together, these molecular findings support the concept that pain in HS is not solely inflammatory but reflects integrated neuroendocrine–immune dysregulation, encompassing circadian, endocrine, and androgenic pathways that collectively shape the pain experience in affected patients.

#### 3.5.2. Therapeutic Interventions Targeting Neuroendocrine Pathways

Therapeutic interventions targeting neuroendocrine pathways span endocrine, metabolic, neural, and immune domains, as summarized in Table 2. Androgens may contribute to HS flares through androgen-induced follicular occlusion secondary to hyperkeratosis. Spironolactone, used as an anti-androgen therapy, has been shown to provide relief for women with HS. Finasteride, which blocks the conversion of testosterone to DHT, decreases the sensitivity of the pilosebaceous unit to androgen stimulation. In a case series of three pediatric patients’ finasteride (initiated at 1.25–5 mg daily and titrated up to 5–10 mg daily based on clinical response) was associated with decreased frequency and severity of flares, with treatment durations ranging from 2.5 to 6 years [73]. Additionally, oral contraceptives (OCP) like ethinylestradiol/cyproterone acetate 50 mg and ethinylestradiol 50 micrograms/norgestrel 500 micrograms reduce active circulating androgens. The use of these OCP for 12 months resulted in 29% of women clearing their HS [74]. Emerging evidence suggests that prolactin may contribute to immune activation in HS, as it can function as an immunomodulatory cytokine by activating autoreactive B cells, modulating T cell responses, and amplifying pro-inflammatory signaling [21]. Clinically, patients receiving medications associated with hyperprolactinemia (e.g., antipsychotics, lithium) have been reported to experience more frequent HS exacerbations [6]. However, prolactin-targeted therapies are not currently established in HS management, and its role remains primarily mechanistic rather than therapeutic. Recognition of hyperprolactinemia may therefore inform individualized management in selected patients, rather than representing a primary treatment strategy. Another anti-androgen therapy employed in HS is cyproterone acetate (CPA). CPA is a competitive antagonist of testosterone and DHT and an agonist of the progesterone receptor. Additionally, CPA lowers LH and FSH levels. Together this results in a strong anti-androgenic effect and is helpful in the treatment of HS [5]. Mindfulness and exercise have been shown to improve mental health outcomes of the disease. Mindfulness has been proven to be a successful mitigator of anxiety and therefore helpful in reducing the comorbid anxiety often experienced with HS [75]. Other stress management techniques such as relaxation exercises and cognitive behavioral therapy have also shown promise [44]. Given the high prevalence of metabolic dysfunction in HS, increasing attention has been directed toward insulin-modulating therapies. Recent literature, including emerging data on GLP-1 receptor agonists, suggests potential benefit beyond weight reduction, possibly through modulation of insulin resistance, adipokine signaling, and inflammatory pathways [76]. A recent systematic review reported that GLP-1 receptor agonists, including liraglutide and semaglutide, were associated with reductions in weight, systemic inflammatory markers, and lesion severity in HS patients, with proposed mechanisms involving suppression of TNF-α, IL-17, and NF-κB signaling pathways [36]. New emerging drugs targeting small molecules are currently being developed and/or in active clinical trials. PTM-001, an investigational small-molecule inhibitor targeting interleukin-1 beta (IL-1β), is currently in phase 2 clinical trials and has been associated with reductions in IL-1β signaling. Additionally, emerging small-molecule therapies such as upadacitinib, a selective Janus kinase 1 (JAK1) inhibitor; LYS006, an inhibitor of the NLRP3 inflammasome; LOU064, an anti-interleukin-17 (IL-17) monoclonal antibody; and RGRN-305, a heat shock protein 90 (HSP90) inhibitor, have demonstrated improvements in clinical endpoints, including reductions in Hidradenitis Suppurativa Clinical Response 50% (HiSCR-50) scores (Table 2) [77].

## 4. Research Gaps and Future Directions

Despite growing recognition of neuroendocrine–immune interactions in HS, substantial knowledge gaps remain. Longitudinal biomarker studies are urgently needed to characterize temporal relationships between hormonal fluctuations, stress biomarkers, neuropeptide levels, and disease activity, including both systemic markers (e.g., circulating cortisol, prolactin, cytokines) and tissue-based markers (e.g., lesional neuropeptide expression, immune cell infiltration).

Similarly, serial measurement of sex hormones, prolactin, and adipokines across menstrual cycles in women with HS could predict perimenstrual flares and identify patients most likely to benefit from hormonal interventions. Importantly, future studies should incorporate standardized disease severity measures (e.g., Hurley stage, HiSCR) and anatomical distribution of lesions, as symptom burden, particularly pain and pruritus, may vary by disease stage, location, and sex. Interventional studies examining combined hormonal, immunomodulatory, and stress-reduction therapies may reveal synergistic benefits not achievable with single-modality treatments, particularly when evaluated using clinically meaningful outcomes such as pain reduction, which has been reported in retrospective analyses of HS therapies. Investigation of neuropeptide expression patterns in HS lesions and exploration of neuropeptide-targeted therapeutics represent particularly promising research directions. For example, immunohistochemical characterization of substance P, CGRP, and VIP in HS tissue, stratified by disease severity (e.g., Hurley stage), lesion location, and patient sex, combined with assessment of nerve fiber density and neurogenic markers, and correlated with clinical measures of pain and pruritus, could establish the foundation for trials of neurokinin receptor antagonists or CGRP-blocking agents that have shown efficacy in other pain conditions, while accounting for known fluctuations in disease activity related to hormonal cycles and stress. Given the known variability in HS disease activity and symptom expression, including fluctuations related to hormonal cycles and stress, longitudinal and stage-stratified analyses will be essential to accurately define these relationships. Additionally, mechanistic studies should examine whether stress-reduction interventions (mindfulness-based stress reduction, cognitive behavioral therapy) measurably alter cortisol dynamics, neuropeptide expression, or inflammatory markers in HS patients, providing biological validation for psychosocial approaches currently supported only by symptom-based outcomes.

Building on the mechanistic domains discussed throughout this review, we propose an integrated neuroendocrine–immune model of HS in which hormonal signaling, stress-mediated HPA axis dysregulation, neuropeptide release, and immune activation operate within a unified, bidirectional network rather than as isolated pathways (Figure 1). In this model, genetic predisposition and environmental triggers initiate follicular occlusion and rupture, releasing DAMPs and PAMPs that activate innate immunity. Hormonal factors, particularly androgens, prolactin during stress, and metabolic hormones in the setting of obesity, modulate the intensity and persistence of this inflammatory response. Chronic inflammation and the psychosocial burden of disease activate the HPA axis, leading initially to elevated cortisol but ultimately to glucocorticoid resistance and impaired anti-inflammatory control. Stress and inflammatory mediators trigger release of neuropeptides from cutaneous nerve endings, which further amplify immune activation and generate the pain and pruritus that define the patient experience. These symptoms, in turn, perpetuate psychological stress, maintaining HPA axis activation and neuropeptide release. This reciprocal, self-reinforcing loop underscores the need for a network-based conceptual model that explicitly depicts bidirectional communication among endocrine, neural, and immune systems.

## 5. Discussion

The neuroendocrine–immune model of HS pathogenesis represents a fundamental reconceptualization of this disease, moving beyond a purely dermatologic perspective to embrace its nature as a systemic disorder involving coordinated dysfunction of hormonal, neural, and immune systems. The evidence synthesized in this review demonstrates that HS cannot be adequately explained by follicular occlusion and immune activation alone. Rather, the characteristic features of HS, including perimenstrual flares, stress-induced exacerbations, severe pain and itch disproportionate to visible inflammation [78], and associations with metabolic syndromes all point toward integrated neuroendocrine–immune dysregulation as a central pathogenic mechanism.

The clinical heterogeneity of HS in terms of disease severity (e.g., Hurley stage), lesion location, pain burden, and sex-specific differences may reflect variability in the relative contributions of different components of the neuroendocrine–immune network. Some patients may exhibit predominantly androgen-driven disease, explaining the efficacy of anti-androgen therapy in selected cases. Others may demonstrate stress-amplified pathology characterized by HPA axis dysregulation and enhanced neuropeptide signaling, potentially benefiting from stress-modulating or neurogenic-targeted strategies. Subset of patients may have metabolic hormone dysregulation as a dominant driver, with insulin resistance and adipokine imbalance perpetuating inflammatory cascades, making them candidates for therapies that improve insulin signaling, such as metformin or GLP-1 receptor agonists. This heterogeneity reflects the need for precision medicine approaches that stratify patients according to neuroendocrine profiles rather than relying solely on inflammatory phenotype.

Current HS management focuses primarily on immune suppression (biologics targeting TNF-α, IL-17, or IL-12/23), which have been shown to improve not only inflammatory lesion burden but also patient-reported pain outcomes in clinical trials, and surgical excision of affected tissue [79,80,81]. While these approaches address downstream inflammatory consequences, they do not target the neuroendocrine triggers and amplifiers that may drive disease initiation and flares.

Paradoxical inflammatory reactions associated with biologic therapy further highlight the complexity of immune dysregulation in HS. Case series of HS patients treated with adalimumab have described development of paradoxical psoriasiform eruptions despite initial improvement in underlying HS activity [82]. Reported patients were predominantly overweight or obese individuals with severe Hurley stage III disease and long-standing HS, with some demonstrating family histories of psoriasis. Proposed mechanisms include dysregulated type I interferon signaling following TNF-α blockade, suggesting that targeted immune suppression may unintentionally activate alternative inflammatory pathways in susceptible individuals. These findings further support the concept that HS pathogenesis involves heterogeneous and interconnected immune networks rather than isolated inflammatory mediators.

The neuroendocrine–immune model suggests that optimal management requires combination strategies: biologics to control acute inflammation, hormonal therapies to modulate androgen and prolactin effects, metabolic interventions to address insulin resistance and adipokine dysregulation, and psychosocial interventions to interrupt stress-inflammation feedback loops. Such multifaceted approaches align with the complex, systemic nature of HS pathogenesis revealed by this framework.

Several limitations of the current evidence base warrant acknowledgment. First, much of the data linking hormonal and stress pathways to HS are observational, limiting causal inference. In addition, despite widespread clinical use of hormonal therapies such as oral contraceptives, controlled clinical trials evaluating their efficacy in HS remain limited. Studies directly evaluating HPA axis function in HS remain sparse, and portions of the stress-axis discussion are extrapolated from broader chronic inflammatory literature. Third, neuropeptide expression in HS lesions has not been systematically quantified, constraining our ability to directly link neurogenic signaling to pain and pruritus in this population. Additionally, portions of the proposed neuroendocrine–immune framework remain hypothesis-generating, as direct mechanistic evidence in HS is still limited for several pathways, including HPA axis dysfunction and neuropeptide-mediated signaling. Fourth, most available studies examine endocrine, neural, or immune pathways independently rather than simultaneously, limiting insight into temporal and mechanistic interactions among systems. Limited quantitative and longitudinal HS-specific data restrict the ability to determine the clinical effect magnitude and translational relevance of several proposed neuroendocrine–immune pathways. Finally, clinical trials explicitly testing integrated neuroendocrine–immune therapeutic strategies have not yet been performed.

Importantly, these limitations reflect gaps in the broader evidence base rather than shortcomings of this review. Continued mechanistic and translational research will be required to determine whether the integrated model proposed here yields measurable improvements in patient outcomes.

## 6. Conclusions

This review proposes a neuroendocrine–immune framework for understanding HS, emphasizing that pain and pruritus arise from integrated hormonal, stress-mediated, neural, and immune dysregulation rather than inflammation alone. Sex hormones, prolactin, metabolic dysfunction, HPA axis alterations, and neuropeptide signaling interact within self-reinforcing feedback loops that sustain inflammation and amplify symptom burden.

Clinically, this model supports a shift beyond isolated immune suppression toward multidimensional management incorporating hormonal modulation, metabolic intervention, stress-targeted therapies, and cytokine-directed treatments. Precision approaches that account for neuroendocrine heterogeneity may improve patient stratification and therapeutic outcomes.

Future research should prioritize integrated biomarker studies and trials targeting multiple nodes within this network. Advancing HS care will require multidisciplinary collaboration and continued investigation into the interconnected neuroendocrine–immune mechanisms driving this disease.

## Figures and Tables

**Table 1:** Summary of literature search strategy for narrative review on neuroendocrine–immune signaling in HS.

**MeSH Terms**	Hidradenitis Suppurativa; Neuroendocrine System; Stress, Physiological; Hypothalamo-Hypophyseal System; Adrenal Cortex Hormones; Androgens; Corticotropin-Releasing Hormone; Cytokines; Pain Perception; Pruritus; Immune System Phenomena; Inflammation Mediators; Metabolic Syndrome; Obesity; Sex Hormones
**Keywords**	Hidradenitis suppurativa; acne inversa; neuroendocrine–immune axis; HPA axis; stress response; cortisol; prolactin; androgens; estrogen; metabolic dysfunction; insulin resistance; adipokines; IL-17; TNF-α; neuropeptides; CGRP; substance P; pain; pruritus; psychosocial stress; hormonal regulation; chronic inflammation; therapeutic targets; biologics; hormonal therapy
**Databases Searched**	PubMed, Scopus, Web of Science
**Date Range**	January 1990–September 2025
**Study Selection Approach**	Studies were selected based on relevance to neuroendocrine–immune mechanisms in HS, with emphasis on pathways related to pain, pruritus, hormonal signaling, stress-axis dysfunction, and immune modulation
**Language and Filters**	English language only; human and mammalian studies; article types limited to original research articles, systematic reviews, meta-analyses, and narrative reviews
**Inclusion Criteria**	1. Peer-reviewed primary studies or reviews focusing on HS pathogenesis or related inflammatory skin diseases. 2. Articles examining hormonal, neuroendocrine, metabolic, or stress-related pathways in HS or comparable immune-mediated dermatoses. 3. Studies investigating cytokines, neuropeptides, endocrine hormones, or HPA axis signaling relevant to HS. 4. Experimental, clinical, or translational work linking endocrine or neural factors to immune modulation, pain, or itch in HS.
**Exclusion Criteria**	1. Non-peer-reviewed sources or conference abstracts. 2. Articles limited to surgical or procedural management without mechanistic insight. 3. Studies unrelated to neuroendocrine or immune mechanisms (e.g., microbiome-only or imaging-only studies).
**Additional Sources**	Reference lists of included publications, recent meta-analyses on HS immunopathogenesis, and manual searches of journal special issues
**Review Design**	Narrative review with thematic synthesis; formal meta-analysis and PRISMA-guided risk-of-bias assessment were not performed due to heterogeneity in study design and outcome measures
**Final Studies Included in Review**	73

**Table 2:** Therapeutic strategies in hidradenitis suppurativa mapped to the neuroendocrine–immune model. Summary of current and emerging therapeutic approaches in HS organized according to the neuroendocrine–immune framework proposed in this review. Treatments are categorized by their primary domain of action (endocrine, metabolic, neural/HPA axis, or immune) and aligned with their proposed mechanistic effects on inflammatory signaling, hormonal modulation, stress-axis regulation, and symptom generation, including pain and pruritus. While some therapies primarily target downstream immune mediators, others may exert indirect effects on neurogenic inflammation and endocrine dysregulation. Emerging neuropeptide-targeted strategies remain investigational and are included to illustrate potential future directions.

Therapeutic Category	Example Agents	Targeted Domain	Proposed Mechanism Relevant to Pain/Pruritus
Anti-androgen therapy	Spironolactone, Finasteride, Cyproterone acetate	Endocrine	Reduces androgen-driven follicular occlusion and inflammatory priming
Hormonal stabilization	Combined OCPs	Endocrine	Modulates estrogen–androgen balance and inflammatory tone
Prolactin modulation (indirect)	Adjustment of hyperprolactinemia-inducing medications	Endocrine/Immune	Reduces prolactin-mediated immune activation
Metabolic therapy	Metformin, GLP-1 receptor agonists	Endocrine–Metabolic	Improves insulin resistance and adipokine imbalance; reduces systemic inflammation
Stress-targeted interventions	CBT, mindfulness, exercise	Neural–HPA Axis	May reduce stress-induced neuropeptide release and HPA dysregulation
Cytokine-targeting biologics	TNF-α inhibitors, IL-17 inhibitors, IL-1 inhibitors	Immune	Reduces inflammatory mediators that sensitize nociceptors and pruriceptors
Emerging neuropeptide-targeting therapies	NK-1 antagonists, CGRP-targeting agents (theoretical)	Neural	Potential modulation of neurogenic inflammation and symptom signaling
Systemic corticosteroids	Prednisone	Immune	Broad immunosuppression with rapid reduction in pro-inflammatory cytokines (e.g., TNF-α, IL-1β), leading to decreased nociceptor sensitization and acute improvement in pain and inflammatory flares

## Data Availability

No new data were created or analyzed in this study.

## Abbreviations

The following abbreviations are used in this manuscript:

HS	Hidradenitis Suppurativa
PMID	PubMed Identifier
MeSH	Medical Subject Headings
ELISA	Enzyme-Linked Immunosorbent Assay
HPA axis	Hypothalamic–Pituitary–Adrenal axis
CRH	Corticotropin-Releasing Hormone
ACTH	Adrenocorticotropic Hormone
MC2R	Melanocortin 2 Receptor
IGF-1	Insulin-Like Growth Factor-1
BMI	Body Mass Index
TNF-α	Tumor Necrosis Factor alpha
IL-1β	Interleukin-1 beta
IL-6	Interleukin-6
IL-17	Interleukin-17
IL-36	Interleukin-36
G-CSF	Granulocyte Colony-Stimulating Factor
PGE2	Prostaglandin E2
DAMPs	Damage-Associated Molecular Patterns
PAMPs	Pathogen-Associated Molecular Patterns
Th1	T helper type 1 cell
Th17	T helper type 17 cell
Treg	Regulatory T cell
CGRP	Calcitonin Gene-Related Peptide
VIP	Vasoactive Intestinal Peptide
OCP	Oral Contraceptive Pill
DHT	Dihydrotestosterone
CPA	Cyproterone Acetate
LH	Luteinizing Hormone
FSH	Follicle-Stimulating Hormone
SHBG	Sex Hormone-Binding Globulin
POMC	Proopiomelanocortin
IL-12/23	Interleukin-12/23 (cytokine complex)
HISCR-50	Hidradenitis Suppurativa Clinical Response 50%
